# Radiological Activity Score (RAS)—MRI Characteristics in Dysthyroid Optic Neuropathy in a Multi‐Ethnic Thyroid Eye Disease Population

**DOI:** 10.1111/cen.15272

**Published:** 2025-05-20

**Authors:** Malik Moledina, Vickie Lee, Kunwar Bhatia, Gitta Madani, Claire Feeney, Nicole George, Nour Houbby, Daisy Metcalf, Natalie Man, Rajni Jain, Ahmad Aziz, Ravi Kumar Lingam

**Affiliations:** ^1^ Oculoplastics and Adnexal Service, Western Eye Hospital Imperial College Healthcare NHS Trust London UK; ^2^ Imperial College Ophthalmology Research Group (ICORG) London UK; ^3^ Department of Radiology Imperial College Healthcare NHS Trust London UK; ^4^ Department of Metabolic Medicine St Mary's Hospital, Imperial College Healthcare NHS Foundation Trust London UK; ^5^ Department of Metabolism, Faculty of Medicine, Digestion and Reproduction Imperial College London London UK; ^6^ National Institute of Health Research Imperial Clinical Research Facility London UK; ^7^ Department of Radiology London North West University Healthcare NHS Trust Harrow UK

**Keywords:** dysthyroid optic neuropathy, Graves orbitopathy, Graves' ophthalmopathy, thyroid eye disease, thyroid orbitopathy

## Abstract

**Purpose:**

Dysthyroid Optic Neuropathy (DON) is a sight threatening and diagnostically challenging complication of Thyroid Eye Disease (TED). We provide a comparative analysis of the MRI features associated between patietnts with and without DON.

**Methods:**

Anonymised retrospective cohort study of patients with TED over eleven years. All patients had Axial T1‐weighted and coronal 3 mm MRI STIR images. In a subset, a 3‐mm‐thick non‐echoplanar HASTE DWI sequence was acquired in the coronal plane, and an ADC map was calculated using the diffusion scan raw data. Assessment of apical crowding (AC), extraocular muscle (EOM) enlargement (E), peri‐muscular fat (PMF)/muscle signal intensity (SI) and Apparent Diffusion Coefficient (ADC) were analysed on coronal images.

**Results:**

Twenty‐six consecutive DON and 516 non‐DON cases. In the DON group, elevated EOMSI, PMFSI, EOME and AC were present in 54.6%, 25.9%, 72.7% and 64.6%, respectively, compared to 24.1%, 6.2%, 42.1% and 5.9% in the non‐DON (*p* = 0.001, *p* = 0.000, *p* = 0.001 and *p* = 0.000). The average ADC value in the DON cohort was 1373 ± 319 versus 973 ± 237 in the non‐DON (*p* = 0.000). Likelihood of DON on Univariable Regression Analysis (Odds Ratios): Apical Crowding (29.1 x *p* = 0.000) and ADC Value ≥ 1200 (7.3 x *p* = 0.000). On Multivariable Regression Analysis (Odds Ratios): Apical Crowding 22.1 x (*p* = 0.000) and ADC Value ≥ 1200 3.7 x (*p* = 0.027).

**Conclusion:**

MRI features associated with a higher diagnostic likelihood of DON include significant AC and elevated ADC values. ADC may show reasonable promise in diagnosing and predicting DON.

## 1 Introduction

Thyroid Eye Disease (TED) is an autoimmune orbital inflammatory disease in which 2%–8% of patients develop Dysthyroid Optic Neuropathy (DON), often resulting in irreversible visual impairment. [1, 2] The predominant mechanism involves an orbital compartment syndrome mediated by enlargement of the extraocular muscles (EOM) and inflammation at the orbital apex, resulting in optic nerve ischaemia [2].

Timely recognition and management of DON is critical but can be challenging due to a lack of gold‐standard clinical diagnostic criteria [3]. Controversy still exists on which parameters should be given the greatest weight, particularly when diagnosing early or atypical DON [3].

Magnetic Resonance Imaging (MRI) is a noninvasive imaging modality that plays a pivotal role in the management of TED [4]. Recommended by the EUGOGO guidelines, orbital MRI's superiority in soft tissue visualisation helps evaluate disease activity, gauge the effectiveness of immunosuppressive treatments, and support the classification of disease severity [5, 6]. This is particularly important in cases involving DON, where no single measure or biomarker has been shown to be consistently and reliably to confirm or exclude DON [2]. Several imaging measures have already been shown to be associated with DON and are utilised in risk stratifying patients, surgical planning, elucidating pathophysiology and providing crucial guidance in equivocial or atypical cases [4, 6].

In our study we aim to provide a comparative analysis to understand how some of the traditional imaging measures, which have been previously associated with DON, relate to a multi‐ethnic population and explore some less established MRI markers such Peri‐Musclar Fat Signal Intensity and Apparent Diffusion Coefficient and how these relate to DON in a multi‐ethnic population from a large TED tertiary centre.

## 2 Materials and Methods

An anonymised retrospective cohort study of TED patients within a tertiary centre across two trusts: London North West University and Imperial College NHS Trust. Consecutive patients diagnosed with TED were included between 01/01/2011 and 01/01/2022. Patients were managed in a multidisciplinary clinic under TED‐specialist oculoplastic surgeons (VL, AA, RJ), endocrinologists (CF), along with a team of allied healthcare professionals for visual field (VF), motility assessment and ocular imaging. Patients underwent MRI orbital imaging under three specialised radiologists (KB, RL and GM) with their findings reported in a structured and homogenous manner and who were unaware of the CAS score at the time of reporting. Where any disagreement existed, consensus was reached with a multi‐disciplinary team meeting where all three radiologists were present and a consensus reached.

Data was extracted from a TED database populated contemporaneously following each clinic visit. Prospective data collected at each visit included diagnosis (GH, hypothyroidism, Hashimotos or euthyroid), status (euthyroid, hyper or hypothyroid), activity (CAS), severity (EUGOGO) and treatment. Active hyperthyroidism was an elevated T3/T4 level above the normal range and suppressed TSH. Retrospective data was collected from electronic records by three data collectors (NH, DM, NM) who were blinded to the study aims and objectives and to the long term outcomes of the patients at the time of extraction: demographics (ethnicity, sex and smoking status), clinical parameters (clinical history, vision, RAPD status, colour plates, exophthalmometry, and diplopia scoring) and investigations (VF, MRI parameters from reports: radiological proptosis, STIR signal elevation both in the Extraocular muscles [EOM] and peri‐muscular fat, Apparent Diffusion Coefficient of EOM [ADC‐EOM] values of the most active EOM [7], apical crowding assessment and significant EOM enlargement). Original MRIs were reviewed when required in approximately 10% of cases by a single reviewer (KB). When multiple MRIs were performed before treatment and if there was a change between scans, these would be classed as separate data points. If a patient with DON had a relapse and had a repeat MRI, this, too, would be classed as a distinct data point.

Inclusion criteria: age ≥ 18 years, a clinical diagnosis of TED with ≥ 6 months follow‐up. Exclusion criteria: An MRI performed following definitive surgical treatment or not performed within 6 months of the clinical diagnosis of DON. External Endocrinology input and/or diagnosis of TED/DON in doubt, and/or significant co‐existing ocular or general pathology, which could distort outcome measures, and/or insufficient data.

DON was defined using modified criteria as per Khong and Dayan et al. [8, 9] DON was diagnosed by **two** of the following criteria: (1) Reduced colour vision, (2) Reduced visual acuity of ≥ 1 line or ≥ 5 letters, (3) The presence of a Relative Afferent Pupillary Defect (RAPD), (4) optic disc swelling/abnormalities (without other ascribable causes), and (5) VF changes associated with DON (utilising MD, common identifiable VF patterns found in DON [10, 11]) AND **without** other attributable causes. Confounding variables were reviewed, and contributions assessed to visual assessment. Where doubt existed about confounders’ impact on acuity, these cases were excluded [9]. Baseline vision was determined from prior notes or recent assessments from an ophthalmic professional. When diagnosing DON, the authors were cognisant of the formula developed by Callahan et al. and applied the tool where appropriate in conjunction with clinical judgement [12].

## 3 Image Evaluation

**FIGURE 1:**
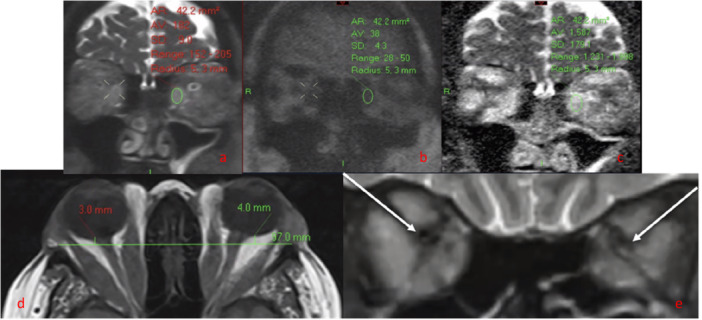
Coronal non‐EPI HASTE DWI MRI images ((a) = b0, (b) = b1000, (c) = ADC map) in a patient with severe TED. A region of interest has been placed in the left medial rectus muscle belly, showing markedly elevated ADC (1587) on (c). (d) Axial MRI T1 image, demonstrating radiological measurement of proptosis‐ the distance between the inter‐zygomatic line and the posterior globe in a TED patient (Normal range: 5.00–10.00 mm posterior to the inter‐zygomatic line [16, 17]). (e) Coronal MRI STIR image showing apical crowding (arrow) of the optic nerves bilaterally in a patient with TED and DON.

MR imaging was performed on a 1.5 T superconductive unit (Magnetom Avanto; Siemens) using a standard Head Matrix coil. Patients obtained T1‐weighted axial and coronal 3 mm STIR images without godalinium contrast (TR = 5640 ms; TE = 91 ms; matrix = 256 112; FOV = 145 mm). In a subset of patients, a 3‐mm‐thick non‐echoplanar HASTE DWI sequence was acquired in the coronal plane (TR = 900 ms; TE = 118 ms; matrix = 192 86; FOV = 145 mm; 18 averages; EPI spacing = 6.28 ms; bandwidth = 465 Hz/pixel; b factors, 0 and 1000 s/mm^2^). Following the acquisition, the scanner software automatically generated an ADC‐EOM map on coronal imaging. The STIR, b = 0, and b = 1000 diffusion‐weighted images were copy‐referenced to ensure the same section position to allow optimal image evaluation and measurement. Utilising the b = 0 image, freehand regions of interest were contoured within the inner border of all active extraocular recti muscles. The extraocular recti muscle with the highest ADC value was utilised. Oblique muscles were not included due to reliability concerns of measurement in an oblique plane. (Figure 1) [7]. Proptosis was calculated utilising an axial T1‐weighted scan and measured using the perpendicular distance from the inter‐zygomatic line to the posterior surface of the globe (Figure 1). Two established methods exist for measuring radiological proptosis in relation to the inter‐zygomatic line, without consensus on the most reliable measure [13]. The first is to the anterior surface of the globe, whilst the second, and our preferred choice, is to the posterior sclera [13]. The advantage of measuring to the posterior surface is that the posterior surface of the sclera is always visible, whilst some studies have found it difficult to distinguish between the closed eyelid and the cornea, which may impact the reliability of readings [14]. Furthermore, the literature has shown this method to be highly reliable [15]. Normative readings range between 5 and 10 mm, which is influenced by ethnicity [15, 16, 17].

**FIGURE 2:**
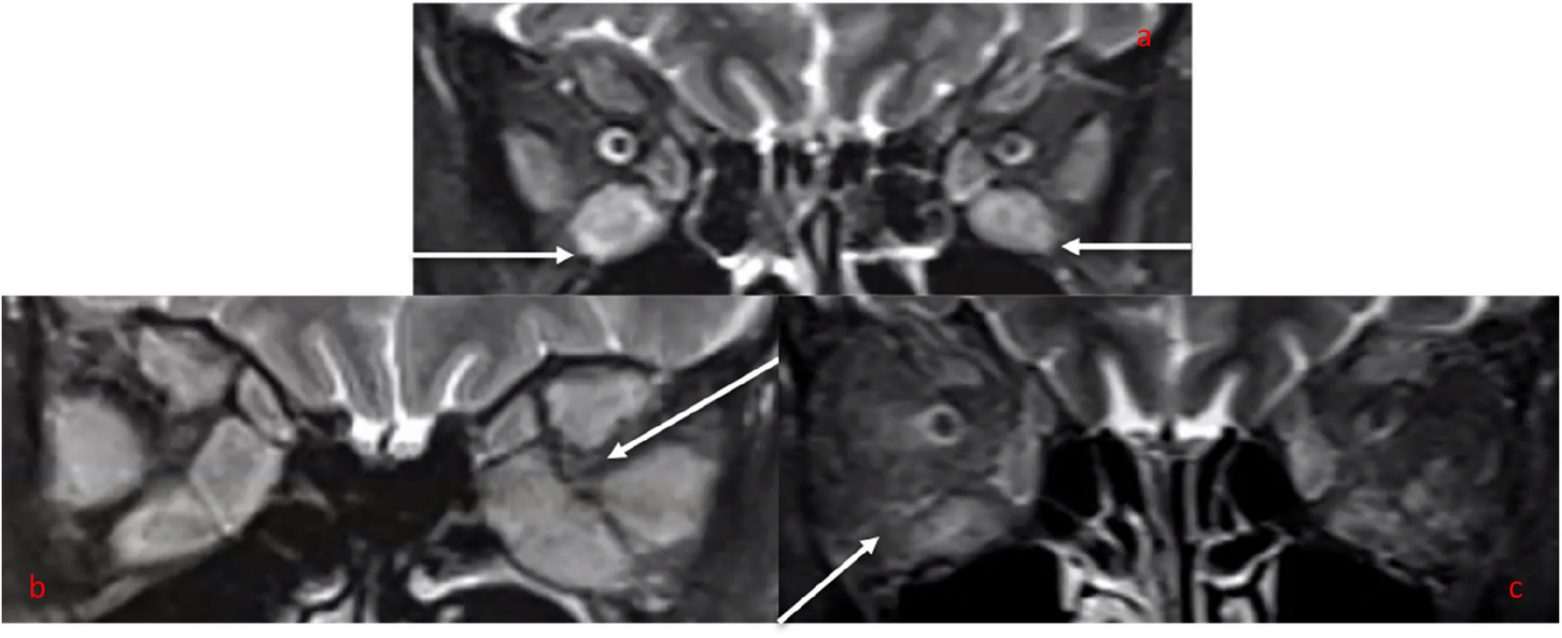
Coronal MRI STIR images (a) Muscle enlargement with elevated signal intensity in the inferior rectus muscles bilaterally in a patient with TED and no‐DON. (b) gross enlargement of all extraocular muscles bilaterally in a patient with TED and DON. This patient also has apical crowding (arrow) on the left side. (c): identifying right peri‐muscular fat signal intensity compared to the left orbit, which is less pronounced in a patient with TED.

Subjective assessments of apical crowding on MRI were as described by Nugent et al. [18] (Figure 1). Apical Crowding (Figure 1), EOM enlargement (Figure 2) and fat/muscle signal intensity utilising a STIR protocol (Figure 2) were analysed on coronal images. Subjective significant EOM enlargement was defined as moderate‐severe muscular enlargement involving ≥ 3 muscles, assessed by the reporting radiologist. Elevated fat/muscle signal intensity was assessed relative to normal tissue, which has previously shown reliable interobserver agreement for the detection of muscle oedema utilising STIR sequences [19].

The study, approved by the institutional audit department was conducted by the tenets of the Declaration of Helsinki.

### 3.1 Statistical Analysis

Two groups were created for analysis: those with DON‐TED and those without. Continuous variables were analysed for normality using a histogram plot, Shapiro‐Wilk and Kolmogorov‐Smirnov Tests. Where normality existed, an independent t‐test was performed; otherwise, Mann‐Whitney U Test was used.

Categorical data were compared using Pearson's Chi‐Square or Fisher's exact test (FET). When a variable was < 5, FET was performed. Univariable binary logistic regression analyses were performed on features where differences were found to be statistically significant (*p* < 0.05) and determined likely to be associated with DON. Multivariable binary logistic regression analysis was calculated on features where differences were found to be statistically significant (*p* < 0.05) and determined likely to be associated with DON on Univariable binary logistic regression analysis. Analysis was performed with SPSS V.24.0 (IBM SPSS for Macintosh).

### 3.2 Missing Values

The retrospective design of this study carries an inherent risk of incomplete data, particularly when assessing data over an extended period. Within our data sample, approximately 16.8% of individual data values were found to be missing over the period analysed where original scans were unavailable to review. An analysis of the missing data patterns suggested the data were Missing at Random (MAR), meaning that the missing data were statistically significantly associated with observed variables when tested. The MAR assumption supports using multiple imputations; five imputed datasets were used to handle the missingness of data for the binary logistic regression analysis. This will limit the introduction of significant bias into the analysis compared to if we had conducted a complete case analysis of our data [20].

## 4 Results

Twenty‐six cases of DON (5.0%) and 516 cases of TED not progressing to DON. The mean follow‐up for the DON group was 30.5 months compared to the Non‐DON group, which was 25.2 months. The MRI reports determined the MRI features, pathology, and content. Ambiguous reports, had original scans re‐reviewed by a specialised consultant radiologist.

### 4.1 Clinical Course, Demographics and Management

33.8% (157/464) of patients were Caucasian, and 22% (110/500) were male. DON patients had an average of 2.73 out of 5 of our DON diagnostic clinical criteria. (Table 1 top)

**TABLE 1:** Patient characteristics of the Don and non‐DON cohorts (top) MRI characteristics of DON and non‐DON TED patients (bottom).

Patient Group				
Patient Characteristics	DON Group (%)	Non DON group(%)	Total N (%)	*p* value
Graves Disease	24/26 (92.3)	469/516 (90.9)	493/542 (91.0)	1.000
Severity	Sight Threatening: 26/26 (100%)	Mild: 282/516 (54.6)		
Severity		Moderate‐Severe: 234/516 (45.3)		
Average duration from Endocrine Diagnosis to DON (Months)	33.1 ± 55.1	—	—	—
Average duration from TED Diagnosis to DON (Months)	13.9 ± 18.80	—	—	—
Average CAS Score	3.52 ± 1.72	0.54 ± 0.80	—	—
Ethnicity	—	—	—	—
Caucasian Patients	5/26 (19.2)	152/438 (34.7)	157/464 (33.8)	0.105
Black Patients	7/26 (26.9)	68/438 (15.4)	75/464 (16.2)	0.164
Asian Patients	5/26 (19.2)	90/438 (20.7)	95/464 (20.5)	0.872
Arab Patients	1/26 (3.8)	16/438 (3.7)	17/464 (3.7)	1.000
Mixed Patients	2/26 (7.7)	8/438 (1.8)	10/464 (2.2)	0.103
Other Patients	6/26 (23.1)	104/438 (23.7)	110/464 (23.7)	0.938
Active Smokers	7/26 (26.9)	115/447 (25.7)	122/474 (25.8)	0.565
Diabetics	7/26 (26.9)	30/362 (8.3)	37/388 (9.5)	0.007
Male Patients	5/26 (19.2)	105/474 (22.2)	110/500 (22.0)	0.474
Prior Radioiodine Use	4/19 (21.1)	66/440 (15.0)	70/459 (15.3)	0.528
Active Hyperthyroidism	9/25 (36.0)	150/509 (29.5)	159/534 (29.7)	0.486
MRI Characteristics	DON Group (%)	Non DON group(%)	Total N (%)	P value
Right Proptosis (mm)	4.58 ± 2.96	5.19 ± 3.24	—	0.408
Left Proptosis (mm)	4.17 ± 3.21	5.45 ± 3.14	—	0.148
Average Proptosis(mm)	4.37 ± 3.05	5.32 ± 3.19	—	0.101
Elevated STIR Signal Intensity Ratio (SIR)	12/22 (54.6)	98/406 (24.1)	110/428 (25.7)	0.001
Superior Rectus	2/8 (25.0)	20/64 (31.3)	22/72 (30.6)	0.535
Medial Rectus	4/8 (50.0)	17/64 (26.6)	21/72 (29.2)	0.220
Inferior Rectus	2/8 (25.0)	20/64 (31.3)	22/72 (30.6)	0.535
Lateral Rectus	0/8 (0.0)	5/64 (7.8)	5/64 (7.8)	1.000
Elevated Perimusclar Fat STIR SIR	7/27 (25.9)	32/518(6.2)	39/545 (7.2)	0.000
Apical Crowding	31/48 (64.6)	30/510 (5.9)	61/558 (10.9)	0.000
Right Orbital Fat Expansion	1/17 (5.9)	25/263 (9.5)	26/280 (9.3)	1.000
Left Orbital Fat Expansion	1/17 (5.9)	24/263 (9.2)	25/280 (8.9)	1.000
Average ADC Value	1373 ± 319	973 ± 237	—	0.000
Signficant EOM Enlargement	24/33 (72.7)	201/477 (42.1)	225/510 (44.1)	0.001
Inferior Rectus Enlargement	24/54 (44.4)	293/801 (36.6)	317/855 (37.1)	0.247
Medial Rectus Enlargement	19/54 (35.2)	198/801 (24.7)	217/855 (25.4)	0.087
Superior Rectus Enlargement	15/54 (27.8)	141/801 (17.6)	156/855 (18.2)	0.061
Lateral Rectus Enlargement	10/54 (18.5)	147/801 (18.4)	157/855 (18.4)	0.976

92.3% (24/26) of DON patients commenced IVMP, which was contraindicated in two patients. Second‐line immunosuppression treatment was initiated 65.4% (17/26) with Mycophenolate, 30.8% (8/26) with Orbital Radiotherapy, and 7.7% (2/26) with Ciclosporin. 53.8% (14/26) of patients required orbital decompression surgery.

### 4.2 Magnetic Resonance Imaging (MRI) Results

Elevated EOM STIR signal intensity (EOMSI), elevated peri‐muscular fat STIR signal intensity (PMFSI), significant EOM enlargement (EOME) and apical crowding (AC) were significantly more likely to be present in the DON cohort. The average ADC value of the most involved *muscle for* the DON cohort was 1373 ± 319 versus 973 ± 237 in the non‐DON cohort (*p* = 0.000) (Table 1 bottom).

### 4.3 ROC Analysis and Odds Ratios

ROC analysis compared ADC‐EOM values, proptosis, AC, EOMSI, EOME and PMFSI for TED patients with DON and those without. All MRI parameters, except ADC‐EOM values, had an area under the curve (AUC) value of < 0.50 and, thus, a low predictive value.

**FIGURE 3:**
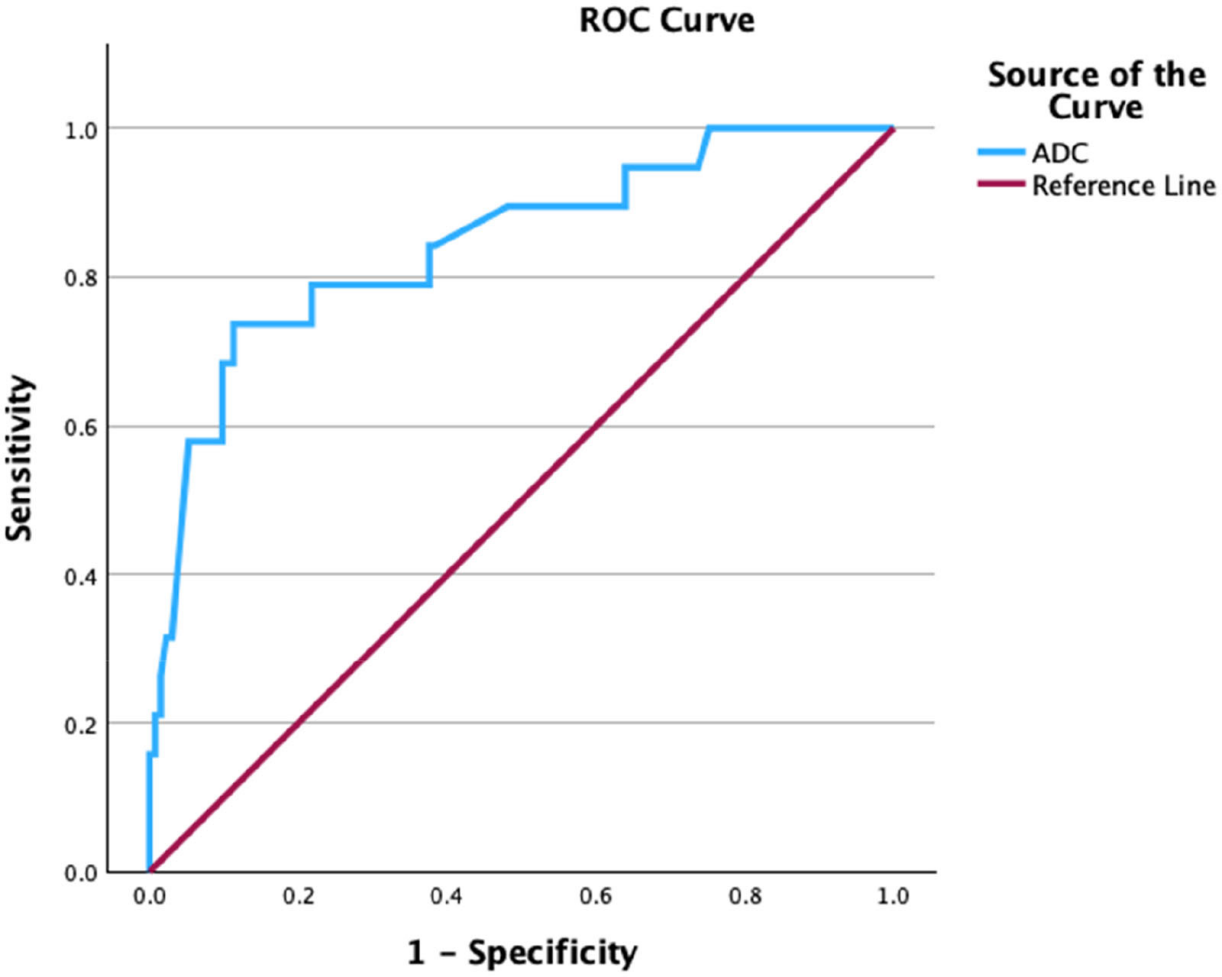
ROC graph to evaluate the utility of ADC values on MRI to predict the likelihood of dysthyroid optic neuropathy. The cohort compared patients with DON to TED patients without DON. ROC curve analysis showed an area under the curve (AUC) of 0.844 (CI: 0.741–0.947 *p* = 0.000). A diagonal line represents a line of no discrimination between disease states.

ROC analysis showed an AUC for ADC‐EOM values of 0.844 (*p* = 0.000). A global accuracy cut‐off value for ADC‐EOM of 1115 (78.9% sensitivity, 78.2% specificity) for DON was selected (Figure 3). Two additional threshold values were selected: ADC‐EOM of 796.5 (100% sensitivity, 24.8% specificity) and ADC‐EOM of 1498 (31.6% sensitivity, 97.7% specificity).

Likelihood of DON on Univariable Regression Analysis (Odds Ratios): Apical Crowding (29.1x *p* = 0.000) and ADC Value ≥ 1200 (7.3 x *p* = 0.000). On Multivariable Regression Analysis (Odds Ratios): Apical Crowding 22.1 x (*p* = 0.000) and ADC Value ≥ 1200 3.7 x (*p* = 0.027) (Table 2)

**TABLE 2:** Univariable and multivariable binary regression analysis of MRI likelihood of having DON.

Univariable Binary Logistic Regression Analysis	Odds Ratio	95% CI	p value
Apical Crowding	29.055	14.451–58.417	0.000
Elevated Muscle STIR Signal Intensity (SI)	2.312	0.374–14.281	0.304
Elevated Perimusclar Fat STIR Signal Intensity (SI)	6.552	0.534–80.365	0.114
ADC Value ≥ 1200	7.313	3.199–16.721	0.000
Signficant EOM Enlargement	2.650	0.805–8.730	0.098

Multivariable Binary Logistic Regression Analysis	Odds Ratio	95% CI	p value
Apical Crowding	22.106	10.690–45.714	0.000
ADC Value ≥ 1200	3.701	1.178–11.631	0.027

## 5 Discussion

Preventing visual loss in DON is predicated on accurate diagnosis and prompt treatment, so assessment methods must be consistent and robust.

The clinical assessment tool, CAS, has limitations and is biased for anterior orbital signs despite some variants of TED where the pathophysiology predominates in a retrobulbar location [21, 22]. This has also been elucidated in other studies. Uddin et al. was able to show TED as a heterogenous group where several variants of DON presented with limited anterior signs such as the 'white eye apex' [23]. Posterior orbital signs are only reflected on follow‐up visits in the CAS score, and thus, over‐reliance on CAS may provide false reassurance. In our study, two of our patients diagnosed with DON had a CAS Score of 1, and interestingly, they had high ADC values of 1800 and 1600, respectively. In addition, 43.7% of our DON patients had a CAS Score < 4 when diagnosed with DON. This is reflected in other studies such as the DON EUGOGO study, where 25% of patients with sight‐threatening disease had a CAS < 3 [24]. It is well recognised that CAS was developed on a Caucasian population and is less reliable in non‐Caucasian groups which is particularly relevant to our study where the majority of our cohort were non‐Caucasian [22] Furthermore, many clinical measurements, some of which input into CAS such as ocular motility and proptosis, are subject to interobserver variability; proptosis is also exophthalmometer dependent [25, 26] Other Functional measurements of ocular function for example, [26]. VF are also subject to variability [27]. It is therefore, prudent for clinicans to be congiscent of low CAS, seemingly 'inactive' patients who may have DON. Therefore, a Radiological Activity Score that targets the pathophysiological site, independent of ethnicity and patient performance, could mitigate the limitations of clinical assessment.

### 5.1 Apparent Diffusion Co‐Efficient of the Most Active EOM Elevation

Diffusion‐weighted imaging (DWI) is a specialised MRI technique that assesses the movement of water protons within tissues as determined by the Apparent Diffusion Coefficient, which provides an ADC value [7, 28]. It is commonly used in various pathologies, such as ischaemic events for example, stroke [29] At our institution, we favour non‐echo planar DWI (non‐EPI‐DWI) rather than a traditional EPI‐DWI. We have found that non‐EP‐DWI is particularly suited for skull base anatomy such as orbits and the temporal bone, now often used to detected conditions such as cholesteatoma, and provides greater image clarity and, consequently, more reliable ADC readings [30]. This can be attributed to improved spatial resolution and reduced artefacts near tissue interfaces, particularly between air and bone [28]. Higher ADC values may represent an underlying inflammatory process due to increased permeability of cell membranes and, consequently, reduced water impedance [31]. It is, therefore, an excellent technique to assess for active inflammation and has been shown to have utility for TED [7, 28]. However, it should be noted that ADC is not specific to TED and may be elevated in various conditions with reduced water proton impedance, such as chronic infarcts, certain benign tumours and other inflammatory processes [29, 30].

Non‐Echo‐planar DWI ADC values are an effective tool in managing and diagnosing TED and provide added benefit through a quantifiable measure of assessment, making comparisons easier and less subjective between participants and different time points [28].

A study from our group showed quantitative evaluation with ADC‐EOM values corresponding well to SI and SIR, which have been conventionally used in detecting disease activity in TED. It was also shown to monitor treatment response in active disease effectively [7]. Our follow‐up study established a strong correlation between ADC‐EOM values, CAS and Severity [28]. In this study, we evaluated the mean ADC‐EOM values of the most involved muscles for 48 patients with active (CAS ≥ 3) disease (cohort 1) compared to nine DON patients. Mean ADC‐EOM values were significantly higher in the DON group than cohort 1 with a 3.7x increased odds of having DON on multivariate analysis when the ADC‐EOM value is ≥ 1200 for any single EOM. This is the first study to analyse the association of DON and elevated ADC‐EOM values using an odds ratio. In this study, an ADC‐EOM value of ≥ 1200 of any EOM had the second highest odds of all factors analysed. Higher ADC‐EOM values may be useful in identifying DON on radiological imaging.

### 5.2 Radiological Proptosis

There was no statistically significant difference between groups. The literature predominantly shows no significant difference in proptosis between DON and Non‐DON groups or that DON groups have greater proptosis [32, 33]. It has been suggested that EOM and adipose expansion may be the primary drivers of higher intraorbital pressures and subsequent optic nerve dysfunction. Although proptosis may act as a compensatory mechanism permitting an anterior decompression of the enlarged orbital volumne, it could also be a decompensatory mechanism in DON by stretching the optic nerves [34].

### 5.3 STIR EOM and Peri‐Muscular Fat SI

SI derived from short tau inversion recovery (STIR) sequence MRI is well‐established in assessing and monitoring TED, and STIR EOMSI is shown to correlate with CAS [35, 36].

Differences in SI between DON and non‐DON TED patients have yet to be fully investigated. We found patients with DON are more likely to have an elevated EOMSI relative to non‐DON TED patients (*p* = 0.001). As SI is a marker of EOM inflammation, this finding concurs with other studies where TED disease activity was associated with DON [8]. However, this was not translated to increased odds on univariate binary logistic regression analysis. This could be due to our study using a subjective assessment of STIR Signal Intensity rather than a quantifiable signal intensity ratio, as this would not be practical to calculate in a routine clinical setting due to the labour and computational intensiveness.

Patients with DON were more likely to have elevated STIR PMFSI relative to non‐DON TED patients (*p* = 0.000). A histological study by Chen et al. found that TED patients had a greater propensity for macrophage infiltration into the orbital fat than controls [37]. Another study noted that cross‐sectional areas of EOMs were greater in several scans when measured on STIR than on T1 images. It's believed this difference was due to inflammatory changes crossing the EOM anatomical boundaries and egressing into peri‐muscular fat. Changes in muscle cross‐sectional area from both images correlated with changes in CAS [35]. Higashiyama et al. investigated the role of SI of orbital fat in TED patients utilising STIR sequences. They found peri‐muscular fat signal intensity ratios (SIR) in active TED were significantly greater than in inactive TED and correlated to CAS. They concluded that orbital fat SIRs are a helpful adjunct in evaluating disease activity in TED [38]. This corroborates our study's findings, which show markers of activity and inflammation, such as elevated PMFSI, tend to be, on average, greater in DON patients than non‐DON patients. However, when analysing odds ratios, this was not proven with statistical significance on univariate regression analysis. The missing data could have negatively influenced our findings which could be an explanation for this. High PMFSI could possibly be a valuable adjunct to distinguish between DON and non‐DON patients but should be further investigated in prospective studies for comprehensive evaluation.

### 5.4 EOM Enlargement

EOM enlargement is utilised in the radiological diagnosis of TED and plays a critical role in the pathogenesis of DON due to optic nerve crowding, which may be a marker for mechanical compression and vascular compromise leading to dysfunction resulting in dysfunction [39]. It has been studied using various parameters, including muscle diameters, volumes and cross‐sectional areas [40] We found patients with DON were statistically significantly more likely to have enlarged EOMs which, mirrors other studies [41]. However, we were unable to prove increased odds with univariate regression analysis as it did not achieve statistical significance. Our study did use a subjective assessment of EOM muscle enlargement which may explain our findings. Another study when creating a quantifiable muscle diameter index which summated the maximum diameters of the EOMs found a 1.7x increased odds of developing DON [41].

TED patients have statistically significantly larger maximum EOM areas relative to controls; the same is true for DON versus non‐DON cohorts [42]. Our data corroborates this, with DON patients more likely to have EOM enlargement relative to non‐DON patients (*p* = 0.001). On subgroup analysis, each muscle was larger in patients with DON than those without DON. Despite not achieving significance, the findings corroborate other studies. In particular, the medial rectus (MR) and superior rectus (SR) were found to be enlarged, achieving values close to statistical significance (*p* = 0.087 and *p* = 0.061). These two muscles have been implicated in the pathogenesis and development of DON [43]. A study investigating EOM enlargement in DON identified a quantitative relationship between DON and EOM enlargement, with the MR exhibiting disproportionate changes in those who had developed DON. The authors postulated this could be due to the MR being predisposed to inflammatory changes [42]. Our study showed that MR had the highest STIR SI in DON patients of any muscle, although the sample size was small and did not reach significance. Nevertheless, this may corroborate the hypothesis that the MR is more predisposed to inflammatory changes than other muscles, with consequences for the optic nerve due to its close anatomical proximity [39].

The superior muscle complex (SMC), in particular SR, is implicated in the development of DON [43]. Starks et al. found that most VF defects in DON were inferior and may correspond to the enlarging of the SMC. Furthermore, it was suggested that SR involvement may be predictive of DON [11]. This may be explained by the anatomical considerations of the optic nerve relative to the annulus of Zinn, with its superior portion closest to the EOMs. Enlargement of the SR may risk crowding of the optic nerve and subsequent neuropathy [44].

### 5.5 Apical Crowding (AC)

Patients with AC had a 22.1x increased odds of having DON, comparable to another study which utilised MDCT and found 7.8x increased odds (CI 2.7–23.3). Whilst our subjective odds were higher, MRI has better soft tissue resolution than CT, making differentiation more accurate [40]. When an objective crowding index score was utilised, the odds increased to 97.2 (CI: 19.6–572.9) [40].

65% of DON patients had AC compared to 6% in the non‐DON cohort (*p* < 0.000), lower than other comparative studies (88%–100%) [45]. This may reflect an inherent subjectivity when assessing AC qualitatively. An objective volumetric orbital apex crowding index, has been described but is labour and computationally intensive [46]. In another study, most patients with DON did not have radiological AC but did have an expansion of the intra‐orbital adipose compartment and proptosis with optic nerve stretch. This appeared to be the mechanism of nerve injury rather than a compressive effect of the nerve displayed through AC [32]. The significance of radiologically reported optic nerve stretch causing DON is inconclusive in the peer‐reviewed literature [34]. However, 18%–21% of nerve stretch in laboratory settings can lead to neuropraxia and nerve injury [47].

### 5.6 Receiver Operating Characteristic (ROC) Analysis

No single MRI parameter, except ADC‐EOM values, had good diagnostic predictability when differentiating between DON and non‐DON patients.

Our previous pilot study performed a subgroup analysis evaluating ADC‐EOM as a diagnostic test for DON via generating a ROC curve and found a cut‐off value of > 1154 to provide 100% sensitivity and 96% specificity in diagnosing DON [28]. The cut‐off value was marginally higher than the optimal cut‐off value in this study based on global accuracy (ADC‐EOM = 1115) with markedly lower sensitivity and specificity values (78.9% sensitivity, 78.2% specificity). This disparity may be explained by the smaller cohort of DON patients (*n* = 5) and comparator groups (*n* = 19) in the original study, possibly skewing the results [28]. Given the specificity and sensitivity values above, the ADC‐EOM value in isolation is insufficient to diagnose DON with certainty. As explained earlier, ADC is not specific to TED and is a marker of raised inflammation and may be elevated in other pathologies [29]. Nevertheless, it may provide a useful adjunct in the diagnosis of DON.

Two further threshold values were identified. The first 796.5, patients were unlikely to have DON below this and were deemed low risk. From our data, all patients with TED who presented with DON had an ADC‐EOM value ≥ 796.5. Although this cut‐off is associated with low specificity (24.8%), patients with values below this threshold may be reassured, and other aetiologies should be suspected if visual loss is identified. The second value of 1498 was specific for DON (97.7%). In our practice, patients at this threshold would be alerted to the clinician and closely monitored for the risk of developing DON if the diagnosis was not already apparent. Nevertheless, the low sensitivity value (31.6%) suggests that most patients with DON present with lower ADC‐EOM values.

### 5.7 Limitations

This is a retrospective, non‐blinded study, in which several radiologists with significant experience in TED reported MRI data. We employed several subjective markers, which are standard practice in routine radiological reporting. We did not utilise quantitative radiological measures, with the exception of ADC, to assess EOM disease activity due to their labour and computational intensiveness in routine practice. Our study did have missing values which is common in a retrospective study. We managed data loss by utilising multiple imputation to minimise data loss and bias. Nevertheless, this could have influenced our findings. Future studies could use a prospective analysis and quantitative measures to prospectively evaluate a larger DON cohort with fewer missing values.

## 6 Conclusion

Assessment of TED should be multi‐parametric and we believe that MRI provides an invaluable insight into the pathological process in the deep orbit developing a Radiological Activity Score (RAS) that compliments the Clinical Activity Score (CAS) that is biased for the anterior orbit. RAS will help to overcome the ethnocultural diversity of CAS Scoring. We identified two major MRI features associated with a higher odds likelihood of DON. These were AC and elevated ADC‐EOM. While we cannot advocate treatment based on radiological signs alone these parameters could be used to support the clinical diagnosis of DON and predict DON in a TED population and increase clinical vigilance in the subpopulation of patients with a high RAS.

## Author Contributions

All authors have made substantial contributions to the conception or design of the work or the acquisition, analysis, or interpretation of data for the work; and drafting the work or revising it critically for important intellectual content and final approval of the version to be published.

## Conflicts of Interest

Miss Vickie Lee is the Principal Investigator for the Horizon, Viridian, Lassen and Sling Trials, BOPSS National Lead for Thyroid Eye Disease and has been on the Advisory Board for Horizon/Amgen Argenx and Viridian Pharmaceuticals. Mr Malik Moledina is: Sub Investigator for the Viridian, Horizon and Sling Trial. Dr Nour Houbby is a Sub Investigators for the Horizon Trial. Dr Claire Feeney is a Pfizer employee in an unrelated field since June 2018. This paper was recently presented at ESOPRS Rotterdam 2024, and formed part of the submission that went on to win the ESOPRS Richard Collins Prize 2024.

## Data Availability

Data can be made available on reasonable request.
